# Association of the Neutrophil-to-Lymphocyte Ratio with Lung Function and Exacerbations in Patients with Chronic Obstructive Pulmonary Disease

**DOI:** 10.1371/journal.pone.0156511

**Published:** 2016-06-03

**Authors:** Heock Lee, Soo-Jung Um, Yun Seong Kim, Deog Kyeom Kim, An Soo Jang, Hye Sook Choi, Yee Hyung Kim, Tae Eun Kim, Kwang Ha Yoo, Ki-Suck Jung

**Affiliations:** 1 Department of Internal Medicine, Dong-A University College of Medicine, Busan, Republic of Korea; 2 Department of Internal Medicine, Pusan National University College of Medicine, Busan, Republic of Korea; 3 Department of Internal Medicine, Seoul National University College of Medicine, SMG-SNU Boramae Medical Center, Seoul, Republic of Korea; 4 Department of Internal Medicine, Soonchunhyang University Bucheon Hospital, Bucheon, Republic of Korea; 5 Department of Internal Medicine, Dongguk University Gyeongju Hospital, Gyeongju, Republic of Korea; 6 Department of Internal Medicine, Kyung Hee University Gangdong Hospital, Seoul, Republic of Korea; 7 Department of Internal Medicine, Konkuk University College of Medicine, Seoul, Republic of Korea; 8 Department of Clinical Pharmacology, Konkuk University College of Medicine, Seoul, Republic of Korea; 9 Department of Internal Medicine, Hallym University College of Medicine, Anyang, Republic of Korea; University of Athens, GREECE

## Abstract

**Background:**

The ratio of neutrophils to lymphocytes (NLR) is a widely available marker of inflammation. Several types of inflammatory cells and mediators have been found to be involved in the progression of chronic obstructive pulmonary disease (COPD). We sought to evaluate the association of the NLR with severity of airflow limitation and disease exacerbations in a COPD population.

**Methods:**

We analyzed 885 patients from the Korean COPD Subtype Study cohort that recruited subjects with COPD from 44 referral hospitals. We determined the relationship of NLR levels to severity of lung function using a linear regression model. In addition, we analyzed the experiences of COPD exacerbation according to the NLR quartiles.

**Results:**

NLR levels were inversely associated with severity of airflow limitation as measured by FEV_1_% predicted and absolute values after adjustments for age, gender, body mass index, pack-years of smoking, and the use of inhaled corticosteroid (P<0.001, respectively). In the multivariate binary regression model, the NLR 4th quartile (vs. 1st quartile) was found to be a significant predictor of exacerbations during 1-year follow-up (OR = 2.05, 95% CI = 1.03 to 4.06, P = 0.041). Adding an NLR to FEV_1_ significantly improved prediction for exacerbations during 1-year follow-up as measured by the net reclassification improvement (NRI = 7.8%, P = 0.032) and the integrated discrimination improvement (IDI = 0.014, P = 0.021).

**Conclusions:**

The NLR showed a significant inverse relationship to airflow limitation and was a prognostic marker for future exacerbations in patients with COPD.

## Introduction

Chronic obstructive pulmonary disease (COPD) is a leading cause of morbidity and mortality worldwide, affecting more than 200 million patients [1]. The classic paradigm for the pathogenesis of COPD is an inflammatory response in both the airway and the lung parenchyma to environmental stimuli, most notably cigarette smoking, which culminates in the development of airway obstruction [2]. Recently, there has been increasing evidence that COPD is a multicomponent disease in which systemic inflammation and extra pulmonary manifestations are commonly present [3,4]. In addition, although the severity of airway obstruction, expressed as forced expiratory volume in 1 second (FEV_1_), has guided the diagnosis, assessment, and treatment of COPD, it is now well recognized that FEV_1_ does not adequately represent the complexity of this disease.

Elevated serum concentration of C-reactive protein (CRP), a biomarker of low-grade systemic inflammation, has been associated with impaired lung function and poor prognosis in patients with COPD [5,6]. White blood cell (WBC) count and its subtypes are also well known systemic inflammatory markers. The ratio of neutrophils to lymphocytes (NLR), which is calculated from complete blood count with differential, is an inexpensive widely available marker of inflammation. The availability of the NLR has been demonstrated in the risk stratification of patients with various cardiovascular diseases, many kinds of solid tumors, sepsis, and infectious conditions [7–9]. Gunay et al. reported that NLR values were significantly higher in patients with COPD than in age- and sex-matched healthy control subjects, and these values increased further during acute COPD exacerbations as compared with periods of stability [10].

We herein sought to evaluate the relationship between the NLR and the severity of airflow limitation and to investigate whether the NLR during stable phase have prognostic role on past and future exacerbations in patients drawn from a nationwide COPD registry.

## Methods

### Study population

Patients enrolled in the Korean COPD Subtype Study (KOCOSS) cohort were considered for this analysis. The KOCOSS cohort was a non-randomized, prospective, multicenter observational cohort that consisted of patients with COPD at 44 participating referral centers across Korea who were older than 40 years of age, had a post-bronchodilator FEV_1_/FVC less than 0.7, and had respiratory symptoms such as cough, sputum production, or dyspnea. Patients with any respiratory disease mimicking COPD such as bronchiectasis, asthma, and tuberculosis-destroyed lung were excluded as candidates for the KOCOSS cohort. In addition, patients with myocardial infarction or cerebral infarction within the previous 3 months, collagen vascular diseases, malignant diseases, or inflammatory bowel disease and pregnant women were not registered in the cohort. Any subjects who could not perform spirometry or who had been treated with systemic steroids within the previous 8 weeks were also not listed in the cohort. All eligible patients had been enrolled in the KOCOSS cohort since December 2011. Because the follow-up period varied according to the time of enrollment, we set June 10, 2015, as the cut-off date for available longitudinal data. Patients who did not have a baseline complete blood count were excluded from the current study. In addition, patients whose WBC counts were above 12×10^3^/μL or below 4×10^3^/μL and patients whose NLR was above 20 were excluded owing to possible infection or inflammatory disease. Written informed consent was obtained from all patients. This study was approved by the local institutional review board at each participating center. Each institution participated this study includes Dong-A University, Hallym University, Seoul National University, Pusan National University, Dongguk University, Kyunghee University, Konkuk University, and Soonchunhyang University.

### Baseline evaluation and follow-up

Patients underwent standard spirometry after the administration of 400 μg of inhaled albuterol at each participating center according to ATS/ERS recommendations [11]. The majority of lung function tests were performed with either a SensorMedics Vmax (Yorba Linda, CA, USA) or MasterScreen PFT system (Jaeger, Hoechberg, Germany). A 6-minute walk test was performed to evaluate the functional status of enrolled patients [12]. Patients’ self-reported respiratory symptoms, medications, smoking history, occupational exposure, and coexisting medical conditions were documented at cohort entry, and their subjective dyspnea was objectively assessed with the use of the Modified Medical Research Council (MMRC) Dyspnea Scale [13]. Quality of life was assessed with the COPD Assessment Test (CAT) [14] and St. George’s Respiratory Questionnaire for COPD (SGRQ-C) [15]. Prospective clinical data were gathered through a longitudinal follow-up protocol that was performed every 6 months.

### Neutrophil-to-Lymphocyte Ratio

The total numbers of WBCs, neutrophils, and lymphocytes were measured at each participating center. The NLR was defined as the absolute neutrophil count divided by the absolute lymphocyte count. Neutrophil and lymphocyte values at the time of cohort entry were used to determine NLR values. CRP was not a mandatory laboratory test.

### Study outcomes

The relationship between the NLR and the severity of airflow limitation as measured with spirometry was a primary outcome. Exacerbations during the first year of follow-up and during the previous year were evaluated according to the NLR quartiles or tertiles. Prospective exacerbation data were analyzed for all patients in whom longitudinal follow-up data were available. Data regarding exacerbation history within the past year were gathered at the time of cohort entry. Exacerbation was defined as worsening of one of the respiratory symptoms, such as an increase in sputum volume or purulence or an increase in dyspnea or cough that required treatment with systemic corticosteroids, antibiotics, or both. The occurrence of retrospective or prospective exacerbations was determined at each participating center.

### Statistical analyses

All patients were categorized into the NLR quartiles (Q1≤1.43, 1.43<Q2≤2.04, 2.04<Q3≤2.94, and 2.94<Q4). Continuous data are presented as means ± standard deviation. Categorical data are presented as frequencies and percentages. The linear contrast analysis within the analysis of variance framework or the Jonckheere–Terpstra test was used for trend analyses of continuous data with normal or skewed distribution, when appropriate. The Kolmogorov–Smirnov test was used to determine the normal distribution of continuous variables. Trends in categorical variables were analyzed by the linear-by-linear association. A correlation analysis was performed to determine the relationship between NLR values and airflow limitation, as reflected in the FEV_1_% predicted and absolute values. We log-transformed NLR values to achieve normality and then constructed multiple linear regression models to adjust for the possible confounding effects of age, gender, body mass index (BMI), pack-years of smoking, and the use of inhaled corticosteroid. The multivariate logistic regression analysis was done to determine the association between the NLR and exacerbations during the first year of follow-up. Covariates for the logistic regression model were age, gender, BMI, smoking status, NLR quartile, the presence of exacerbation during the previous year, FEV_1_, and MMRC score. Incremental value of the NLR to the availability of FEV_1_ absolute value to predict future exacerbation was assessed using the net reclassification improvement (NRI) and the integrated discrimination improvement (IDI). The NRI requires a priori meaningful risk categories (we used < 20%, 20% to 50%, and > 50% for the risk of exacerbation during the first year of follow-up). For the NRI and IDI analyses, FEV_1_ absolute value was adjusted with age, gender, and BMI. A P-value ≤ 0.05 was considered statistically significant. All statistical analyses were performed using SPSS 18 software (SPSS Inc., Chicago, IL, USA) and R package version 3.0.2.

## Results

### Patient characteristics

**Fig 1:**
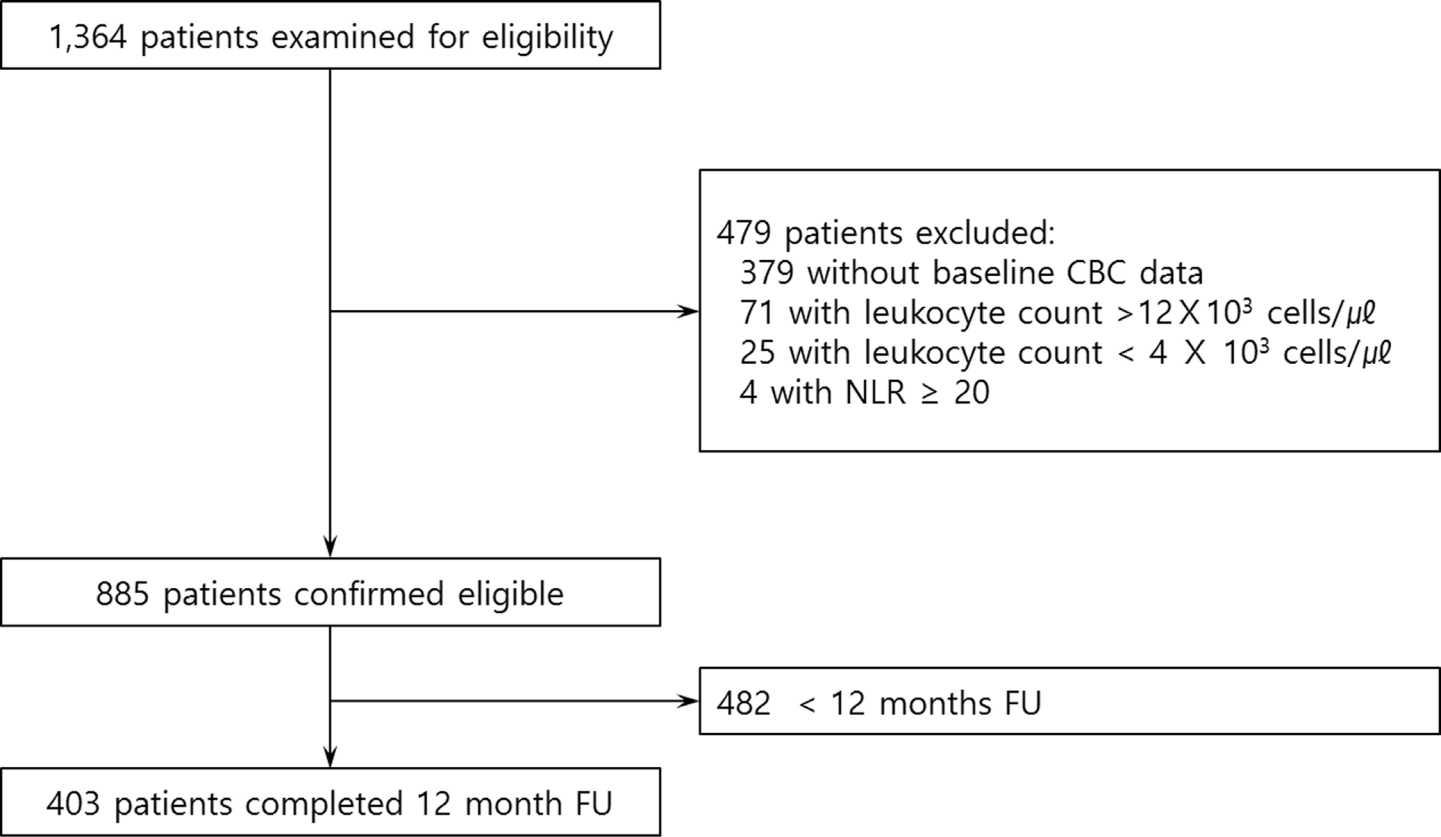
Study flow diagram.

Initially, 1,364 patients recruited from the KOCOSS cohort were eligible for this study. Of this group, 479 patients were excluded, and 885 subjects were included in the current analysis (Fig 1). Mean age was 70.9 ± 7.8 years, and 91.4% of the patients were men. Baseline clinical characteristics are listed in Table 1, categorized according to quartiles based on NLR values. Significant trends included older age (P<0.001) and lower BMI (P = 0.018) in association with higher NLR quartile. Symptoms and quality of life expressed as SGRQ, MMRC, and CAT scores tended to deteriorate as NLR quartile increased (P trend = 0.001, P trend = 0.015, and P trend<0.004, respectively). In addition, participants with higher NLR were more likely to have shorter 6-minute walk distance (6MWD) (P<0.001). As the NLR quartile increased, all lung function parameters deteriorated. Inhaled corticosteroid had been more commonly prescribed in the higher NLR group. There were no significant differences in the incidence of comorbidities among the NLR quartiles.

**Table 1:** Baseline Characteristics of Patients According to Quartiles of Baseline NLR Level.

	Quartile 1	Quartile 2	Quartile 3	Quartile 4	P for trend*
	(n = 222)	(n = 220)	(n = 222)	(n = 221)	
NLR	1.11(0.97–1.33)	1.73(1.57–1.90)	2.41 (2.21–2.59)	4.69 (3.31–4.86)	<0.001
Age, years	69.1 ± 7.4	70.6 ± 7.7	71.7 ± 7.8	72.0 ± 7.9	<0.001
Men	199 (90.0)	192 (86.9)	210 (94.6)	208 (94.1)	0.018
Body mass index, kg/m^2^	23.7 ± 3.2	23.3 ± 3.6	22.7 ± 3.3	22.1 ± 3.3	0.018
Smoking, pack years	38.5 ± 25.3	38.8 ± 26.6	41.5 ± 26.9	39.6 ± 29.3	0.699
Current smokers	67 (30.3)	68 (30.8)	62 (27.9)	50 (22.6)	0.055
Ever smokers	199 (90.0)	191 (86.4)	210 (94.6)	200 (90.5)	0.282
6MWD, meter	398.9 ± 104.2	391.9 ± 109.9	369.9 ± 110.2	349.2 ± 113.8	<0.001
MMRC dyspnea score	1.44 ± 0.99	1.46 ± 0.95	1.63 ± 0.90	1.58 ± 0.91	0.015
SGRQ scale	30.8 ± 17.2	34.7 ± 19.4	34.6 ± 18.2	38.1 ± 21.4	0.001
CAT	14.3 ± 7.2	16.3 ± 8.1	15.4 ± 6.9	16.7 ± 8.1	0.004
ICS use	70 (31.7)	96 (43.4)	91 (41.0)	111 (50.2)	<0.001
Exacerbation during previous 1 year	44 (19.8)	61 (27.6)	53 (23.9)	75 (33.9)	0.004
FEV_1_, % predicted†	60.8 ± 15.4	59.4 ± 17.6	59.1 ± 19.4	53.9 ± 18.9	0.004
FEV_1_, liter†	1.70 ± 0.50	1.57 ± 0.55	1.55 ± 0.50	1.39 ± 0.54	<0.001
FVC, % predicted†	85.9 ± 16.1	84.9 ± 16.4	83.7 ± 19.3	79.3 ± 20.3	<0.001
FVC, liter†	3.28 ± 0.78	3.12 ± 0.81	3.17 ± 0.78	2.90 ± 0.77	<0.001
FEV_1_/FVC ratio, %	51.9 ± 10.2	50.1 ± 12.2	49.5 ± 12.2	47.9 ± 13.2	<0.001
WBC, 10^3^/μL	6.80 ± 1.48	7.06 ± 1.46	7.44 ± 1.73	8.23 ± 1.91	<0.001
Neutrophil, 10^3^/μL	3.01 ± 0.91	3.90 ± 0.91	4.62 ± 1.14	5.94 ± 1.66	<0.001
Lymphocyte, 10^3^/μL	2.77 ± 0.74	2.26 ± 0.52	1.92 ± 0.48	1.40 ± 0.47	<0.001
Comorbidities					
Cardiovascular disease‡	90 (40.9)	83 (39.7)	79 (37.1)	104 (48.1)	0.114
Diabetes mellitus	34 (15.5)	37 (17.7)	34 (16.2)	42 (19.4)	0.377
GERD	26 (11.9)	14 (6.7)	21 (10.0)	21 (9.8)	0.719
Osteoporosis	13 (5.9)	9 (4.3)	10 (4.7)	11 (5.1)	0.782

Values are given as geometric mean (interquartile range) or mean ± SD or numbers (%). CAT, COPD Assessment Test; FEV_1_, forced expiratory volume in 1 second; FVC, forced vital capacity; GERD, gastro-esophageal reflux disease; ICS, Inhaled corticosteroid; MMRC, Modified Medical Research Council; 6MWD, 6 minute walk test distance; NLR, neutrophil to lymphocyte ratio; SGRQ, St. George’s Respiratory Questionnaire; WBC, white blood cell.

*Linear trend from quartile 1 to quartile 4.

†Post-bronchodilator values.

‡Cardiovascular disease incorporates congestive heart failure, myocardial infarction, hypertension and peripheral vascular disease.

### Relationship between the NLR and lung function

**Fig 2:**
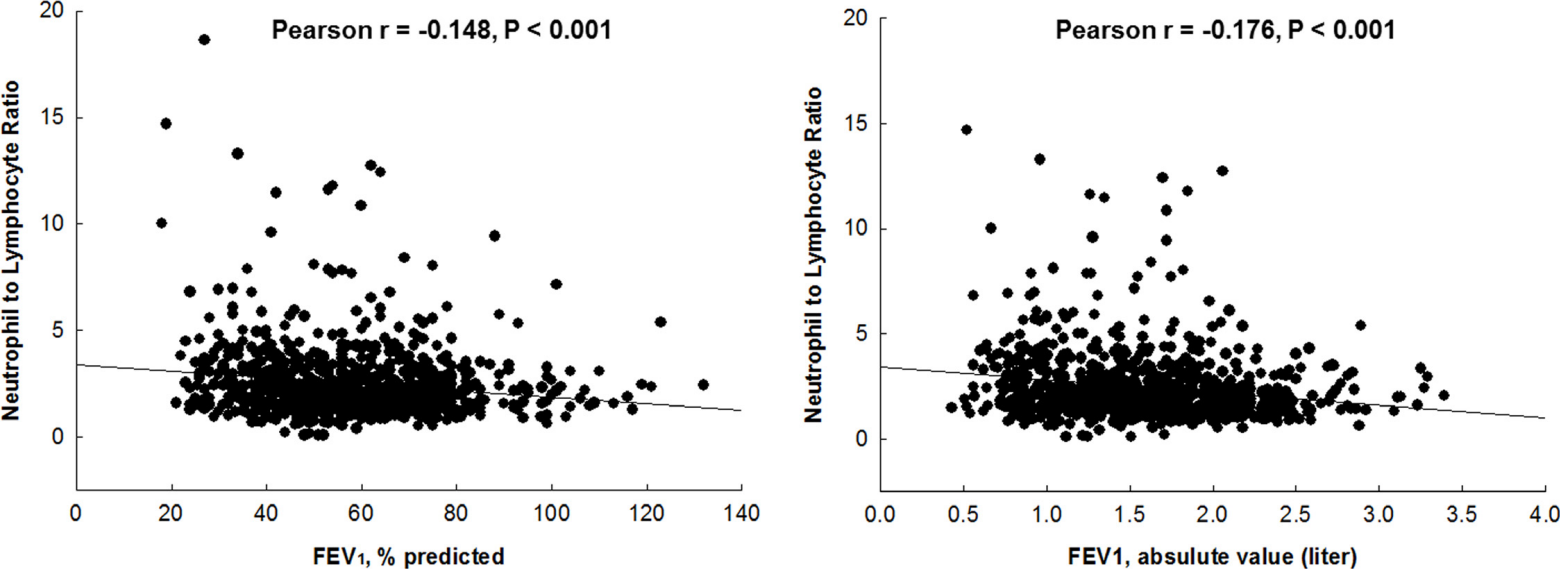
The relationship between neutrophil to lymphocyte ratio and lung function.

NLR levels were inversely associated with the severity of airflow limitation, as measured by FEV_1_% predicted and absolute values (Pearson r = −0.148, P<0.001 and Pearson r = −0.176, P<0.001, respectively) (Fig 2). After adjusting for age, gender, BMI, pack-years of smoking, and ICS use, a significant association between the NLR and the FEV_1_% predicted value and absolute value persisted (Table 2).

**Table 2:** Multivariate Linear Regression Analysis with FEV1 As the Output.

	Unadjusted		Multivariate Model*		
	Effect Estimate (SE)	P-value	ß (SE)	95% Confidence Interval	P-value
**FEV**_**1**_**% Predicted Value**				(R^2^ = 0.054, p <0.001)	
NLR	-1.428 (0.328)	<0.001	-1.316 (0.323)	-1.951 ~ -0.681	<0.001
Smoking, pack years	-0.014 (0.023)	0.537	-0.004 (0.023)	-0.049 ~ 0.040	0.851
Use of ICS	-7.108 (1.233)	<0.001	-6.599 (0.323)	-1.951 ~ -0.681	<0.001
**FEV**_**1**_ **Absolute Value**				(R^2^ = 0.210, p <0.001)	
NLR	-0.050 (0.010)	<0.001	-0.035 (0.009)	-0.053 ~ -0.017	<0.001
Age, years	-0.018 (0.002)	<0.001	-0.016 (0.002)	-0.020 ~ -0.011	<0.001
Men	0.343 (0.064)	<0.001	0.440 (0.065)	0.312 ~ 0.567	<0.001
Body mass index, kg/m^2^	0.039 (0.005)	<0.001	0.035 (0.005)	0.025 ~ 0.045	<0.001
Smoking, pack years	0.001 (0.001)	0.433	0.000 (0.001)	-0.001 ~ 0.001	0.967
Use of ICS	-0.199 (0.037)	<0.001	-0.183 (0.034)	-0.250~ -0.115	<0.001

FEV_1_, forced expiratory volume in 1 second; ICS, inhaled corticosteroid; NLR, neutrophil to lymphocyte ratio; SE, standard error.

*Adjusted with smoking pack year, use of ICS, and NLR for FEV_1_% predicted value and adjusted with age, sex, body mass index, smoking pack year, use of ICS and NLR for FEV_1_ absolute value.

### Relationship between the NLR and exacerbations

**Fig 3:**
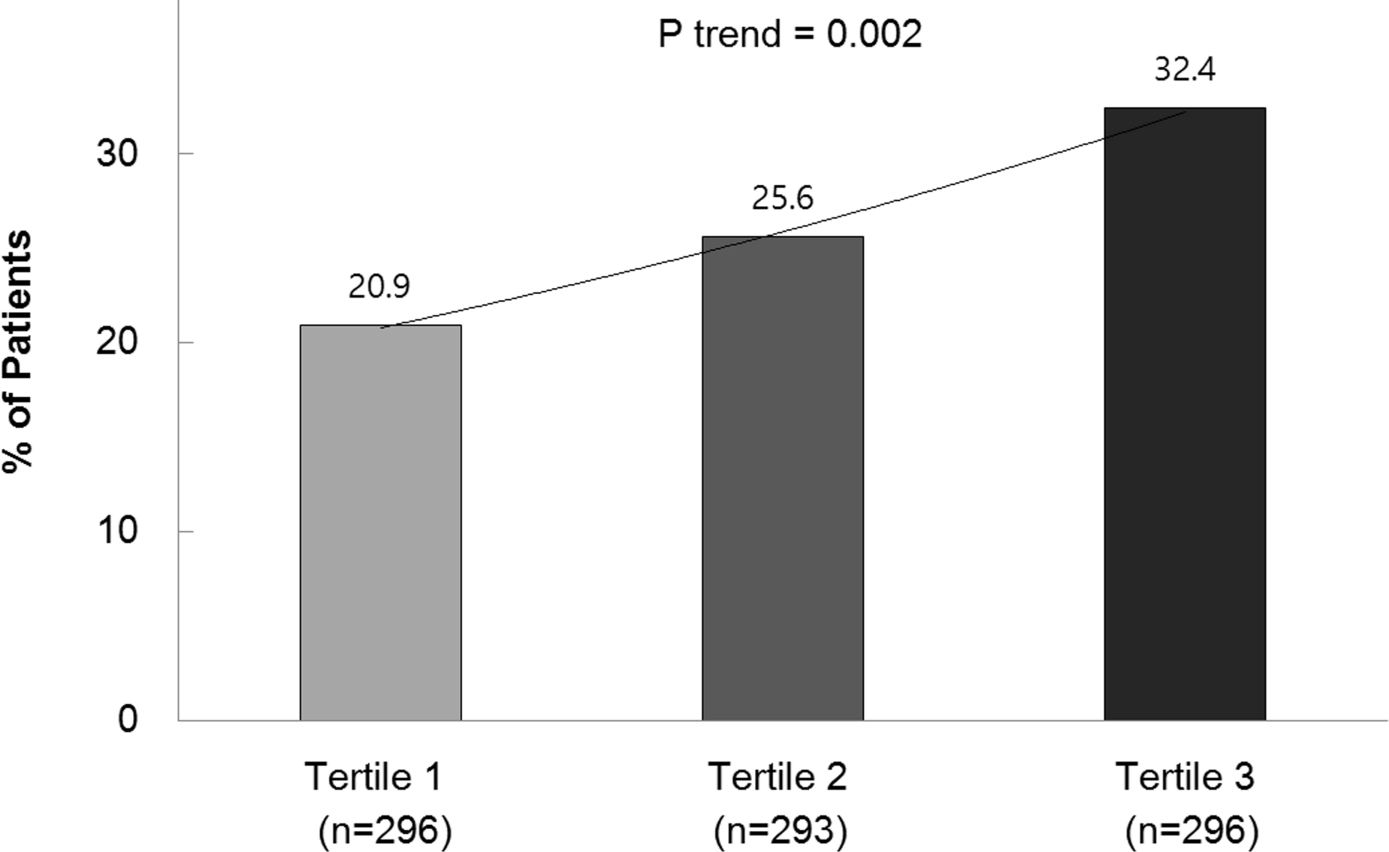
Percentage of patients in each neutrophil to lymphocyte ratio tertile that experienced at least one exacerbation in the previous year.

With regard to COPD exacerbations during the previous year, patients in the 4th quartile of NLR had the highest rate of exacerbations (Table 1). As the NLR tertile increased, exacerbations during the previous year were more frequent (P trend = 0.002) (Fig 3). Out of 885 subjects, 403 patients (45.5%) were available for the 1-year clinical follow-up analysis. On univariate logistic regression analysis, the NLR (per increase of 1 point) was significantly associated with exacerbation in the first year (Table 3). The best predictor of an exacerbation was an MMRC dyspnea score of 2 to 4 (vs. a score of 0 or 1) and the second best predictor was an exacerbation in the year before cohort entry.

**Table 3:** Univariate Association with the Occurrence of Exacerbation during the First Year of Follow-up.

	Odds Ratio (95% Confidence Interval)	P value
**Demographic and clinical factors**		
Age—per 10-year increase	1.37 (1.04–1.82)	0.028
Sex–female vs. male	1.68 (0.77–3.67)	0.196
BMI–per increase of 1 point	1.03 (0.97–1.10)	0.280
Smoking status–current vs. noncurrent	0.69 (0.43–1.10)	0.116
Exacerbation during preceding year	2.91 (1.85–4.58)	<0.001
6MWD–per decrease of 50 meter	1.00 (0.87–1.05)	0.352
**Lung function**		
FEV_1_ –per 5% decrease in % of predicted value	1.13 (1.05–1.20)	<0.001
FEV_1_ –per 100-ml decrease	1.10 (1.06–1.16)	<0.001
FEV_1_/FVC–per 1% decrease	1.04 (1.02–1.06)	<0.001
GOLD stage–per increase to next stage	1.61 (1.18–2.22)	0.003
**Patient-reported outcomes**		
MMRC dyspnea score– 2, 3, or 4 vs. 0 or 1	3.33 (2.18–5.01)	<0.001
SGRQ score–per 4-point worsening	1.14 (1.09–1.19)	<0.001
CAT score–per 1-point worsening	1.06 (1.03–1.09)	<0.001
**Laboratory data**		
WBC–per increase of 1x10^3^/μL	1.07 (0.96–1.20)	0.215
Neutrophil–per increase of 1x10^3^/μL	1.01 (1.00–1.03)	0.022
Lymphocyte–per decrease of 1x10^3^/μL	1.03 (1.00–1.05)	0.080
NLR–per increase of 1 point	1.16 (1.04–1.29)	0.007

BMI, body mass index; CAT, COPD Assessment Test; FEV_1_, forced expiratory volume in 1 second; FVC, forced vital capacity; GOLD, Global Initiative for Chronic Obstructive Lung Disease; 6MWD, 6 minute walk distance; MMRC, Modified Medical Research Council; NLR, neutrophil to lymphocyte ratio; SGRG, St. George’s Respiratory Questionnaire; WBC, white blood cell.

Table 4 lists the factors that were independently associated with exacerbations during the first year of follow-up, as based on the multivariate binary regression model. The NLR 4th quartile (vs. 1st quartile) was also found to be a significant and independent predictor of exacerbations during the first year of follow-up after adjustments for age, gender, BMI, smoking status, NLR quartile, presence of exacerbation during the previous year, FEV_1_, and MMRC score (OR = 2.05, 95% CI = 1.03 to 4.06; P = 0.041). Other factors significantly associated with exacerbations were a history of exacerbations during the previous year, worsening lung function, and a worse score on the dyspnea scale.

**Table 4:** Factors Associated with an Exacerbation During the First Year in the Multivariate Model*.

	Odds ratio (95% CI)	P-value
NLR 4th quartile (vs. 1st quartile)	2.05 (1.03–4.06)	0.041
Exacerbation during previous year	2.12 (1.26–3.54)	0.004
FEV_1_ (per 100-ml decrease)	1.08 (1.02–1.13)	0.009
MMRC dyspnea score– 2 to 4 (vs. 0 or 1)	3.25 (2.05–5.17)	<0.001

FEV_1_, forced expiratory volume in 1 second; MMRC, Modified Medical Research Council; NLR, neutrophil to lymphocyte ratio.

*Adjusted with age, gender, body mass index, smoking status, NLR quartile, presence of exacerbation during previous year, FEV_1_ and MMRC score.

### Discriminative ability of the NLR to predict future exacerbations

The area under the curve of the NLR (AUC = 0.586, 95% CI = 0.528 to 0.643, P = 0.004) was numerically greater than that of the WBC count (AUC = 0.533, 95% CI = 0.475 to 0.592, P = 0.268) and the neutrophil count (AUC = 0.562, 95% CI = 0.504 to 0.620, P = 0.037). In the NRI analysis, net reclassification after adding an NLR to FEV_1_ absolute value in multivariate model placed 7 (4.9%) patients in exacerbation group to higher risk while 7 (2.9%) patients in non-exacerbation group were reclassified into lower risk categories. The overall NRI was 7.8% (p = 0.032) and the IDI was 0.014 (p = 0.021).

## Discussion

In this consecutive series of patients recruited from a real-world COPD cohort, the NLR was found to be a useful marker for assessing disease severity and activity in patients with COPD. The results of this study are the following: (1) NLR levels were inversely related to airflow limitation, as measured by FEV_1_% predicted and absolute values; (2) COPD exacerbations during the previous year were more frequent as the NLR values increased; (3) the highest NLR quartile was a significant and independent predictor of COPD exacerbations during the first year of follow-up; and (4) the availability of FEV1 to predict future exacerbations was improved by incorporating the NLR.

Although there is a complex persistent inflammatory response within the airways and lung parenchyma beginning in the early stages of the disease, COPD is now recognized as a representative systemic inflammatory disease [3,4]. Therefore, it may be reasonable to assume that the level of circulating systemic inflammatory markers is higher in patients with COPD than in healthy control subjects. In fact, several studies have shown that patients with COPD, even during stable periods, have higher values of CRP, fibrinogen, interleukin (IL)-6, and tumor necrosis factor (TNF)-α as compared with those in healthy controls, and elevated levels of these markers of inflammation are associated with an increased risk of disease exacerbations and death [4–6,16,17].

With regard to WBC count and its subtypes, many reports have shown the prognostic value of an elevated WBC count and neutrophilia in the systemic circulation in patients with COPD. In a large Danish COPD population study, an elevated WBC count in combination with elevated levels of CRP and fibrinogen was associated with a two- to four-fold increased risk of major comorbidities such as ischemic heart disease, diabetes, lung cancer, and pneumonia [6]. In two retrospective cohort studies, WBC counts tended to increase in Global Initiative for Chronic Obstructive Lung Disease (GOLD) stages 3 to 4 or GOLD grades C to D, as compared with stages 1 to 2 or grades A to B [6,18]. An elevated WBC count was also associated with increased risk of frequent exacerbations [18,19]. Celli et al. showed that elevated WBC and neutrophil counts were independently and significantly associated with mortality [20]. Although the impact of lymphopenia on chronic inflammatory disease has not been well known, lymphopenia was associated with poor outcomes in patients with an acute infectious disease such as sepsis or bacteremia [21]. In addition, lymphopenia was found to be related to all-cause mortality in patients with COPD [10,22]. It is therefore reasonable to postulate that the NLR, which integrates neutrophilia, an indicator of inflammation, and lymphopenia, an indicator of decreased immune competence/poorer general health, reflects the severity and activity of COPD better than does neutrophilia or lymphopenia alone. In fact, the NLR value was elevated during exacerbation compared with stable state in the same patients [23]. The NLR level was higher in patients with exacerbated COPD than those with stable COPD or control group [24]. However, these studies just showed different NLR levels between stable and exacerbated state. To our knowledge, our study is the first to demonstrate the association between the NLR value during the stable state and past or future exacerbations. Furthermore, we have shown that adding a NLR value to FEV_1_ significantly improves the prediction for future exacerbations. We have also proven that patient reported symptoms or quality of life status deteriorates as the NLR value increases, and exercise capacity also worsens as the NLR level increases. With these findings, we can presume that the NLR may be used as convenient peripheral blood marker to reflect the severity and activity of COPD.

In the current study, the NLR was inversely associated with the severity of airflow limitation. This finding is in agreement with recently published data [23]. The mechanism underlying the relationship between the NLR value and airflow obstruction/exacerbation is not clear. Several studies have shown that neutrophils are a key mediator of the relentless decline in lung function in patients with COPD. When activated, neutrophils released a number of proteolytic enzymes, such as elastase and matrix metalloproteinase, which contributed to the development of emphysema [25]. Airway neutrophilia correlated with the rate of decline in the lung function [26]. Serum levels of calprotectin, another inflammatory marker mainly found in neutrophil granulocytes, were inversely correlated with FEV_1_ [27]. Recently, Milara et al. reported that the peripheral blood neutrophil count was persistently elevated in patients with COPD despite years of smoking cessation [28]. In terms of lymphopenia, previous study showed that many of non-smoking COPD patients had a peripheral blood lymphopenia [29]. Thus the NLR may potentially provide more powerful information regarding airflow limitation or exacerbations. Interestingly, although neither neutrophil nor lymphocyte alone was correlated with FEV_1_ (data not shown), the NLR was correlated with FEV_1_ in our study.

Our study has several limitations. First, this was a non-randomized study, so the possibility of residual confounding from unknown or unmeasured covariates cannot be excluded. Nevertheless, our study was a real-world, prospective, nationwide cohort study that consisted of a relatively large number of patients with COPD who were recruited from nearly all the participating referral centers across Korea. Second, over 90% of the patients included in the study were men and ever smokers. Accordingly, our results may not be applicable to women or never smokers. Third, the association between the NLR and future exacerbations was seen in only half the total cohort population because of the short follow-up period. However, we demonstrated the association between the NLR and the previous year’s exacerbation history in the entire cohort population. In agreement with a previous study [19], past exacerbations was one of the most powerful factors predictive of first exacerbation at 1 year in the current study. Fourth, our study was not enough to support the correlation between the NLR and changes in clinical symptoms and/or signs during clinical course owing to the limited data available. Finally, we could not show the correlation between other serum biomarkers and the NLR because inflammatory markers such as CRP, IL-6, and procalcitonin were not listed as a mandatory laboratory test in our cohort.

In conclusion, the present study shows that the NLR has a significant inverse relationship with the severity of airflow limitation in patients with COPD. Furthermore, the NLR was one of the independent predictors of future exacerbations. Therefore, the NLR, an inexpensive and readily available blood index, may be used as a useful biomarker in patients with COPD.

## Supporting Information

S1 File Data Set. (SAV)

S1 Table Comparisons between original analyses (n = 885) and another analyses including patients excluded owing to the WBC or NLR values (n = 985). (DOCX)
