# Supplementary material for: Association of the Neutrophil-to-Lymphocyte Ratio with Lung Function and Exacerbations in Patients with Chronic Obstructive Pulmonary Disease
Source: PLoS One. 2016 Jun 3;11(6):e0156511. doi: 10.1371/journal.pone.0156511 (PMC4892618; doi:10.1371/journal.pone.0156511)
Supplement: S1 Table — (DOCX) [file pone.0156511.s002.docx]

| Table. Comparisons between original analyses (n=885) and another analyses including patients excluded owing to the WBC or NLR values (n=985) | | | | |  |  |
| --- | --- | --- | --- | --- | --- | --- |
|  | Our analyses (n=885) | | WBC > 12×10^3^/µL or < 4×10^3^/µL and NLR > 20 Included (n=985) | |  |  |
| **Correlation** |  | |  | |  |  |
|  | Pearson r | P value | Pearson r | P value |  |  |
| NLR & FEV_1_ % predicted | -0.148 | <0.001 | -0.171 | <0.001 |  |  |
| NLR & FEV_1_ absolute value | -0.176 | <0.001 | -0.184 | <0.001 |  |  |
|  | | | | |  |  |
| **Past Exacerbations** | N (%) | P trend | N (%) | P trend | % | P value |
| Total no. of patients | 885 (100) | 0.004 | 985 (100) | <0.001 |  |  |
| NLR Q1 | 44 (19.9) |  | 52 (21.2) |  |  |  |
| NLR Q2 | 61 (27.6) |  | 69 (27.9) |  |  |  |
| NLR Q3 | 53 (23.9) |  | 60 (24.5) |  |  |  |
| NLR Q4 | 75 (33.9) |  | 93 (37.5) |  |  |  |
|  | | | | |  |  |
| **Future Exacerbations** | N (%) | P trend | N (%) | P trend |  |  |
| Total no. of patients | 403 (100) | 0.001 | 439 (100) | 0.006 |  |  |
| NLR Q1 | 25 (25.3) |  | 29 (26.4) |  |  |  |
| NLR Q2 | 35 (35.0) |  | 37 (33.9) |  |  |  |
| NLR Q3 | 37 (36.6) |  | 42 (38.2) |  |  |  |
| NLR Q4 | 50 (48.5) |  | 48 (43.6) |  |  |  |
